# Effects of Berberine on Circular RNA Expression Profiles in Human Gastric Cancer Cells

**DOI:** 10.1155/2021/6688629

**Published:** 2021-05-04

**Authors:** Meng Wang, Letao Sun, Li Wang, Yongning Sun

**Affiliations:** ^1^Shanghai Jiao Tong University Affiliated Sixth People's Hospital, Shanghai 200233, China; ^2^Shanghai Jiao Tong University School of Medicine, Shanghai 200025, China; ^3^Gordon F. Derner School of Psychology, Adelphi University, New York 11530, USA; ^4^Shanghai Municipal Hospital of Traditional Chinese Medicine, Shanghai University of Traditional Chinese Medicine, Shanghai 200071, China

## Abstract

**Background:**

Berberine has been demonstrated to have anticancer effects against gastric cancer (GC), but the mechanism of these actions is unclear.

**Objectives:**

To explore the impact of berberine on circular RNA (circRNA) expression profiles in GC and investigate the potential molecular mechanisms associated with circRNAs in GC.

**Methods:**

AGS and HGC27 GC cells were treated with various concentrations of berberine. Cell viability was measured using a Cell Counting Kit-8 assay. Cell proliferation was measured using a cell colony formation assay. Cell apoptosis was measured using flow cytometry. The mitochondrial membrane potential (Δ*ψ*m) was determined using a JC-1 probe. RNA-seq was performed to identify circRNA expression profiles in AGS cells after berberine treatment. Selected differentially expressed (DE) circRNAs were verified using RT-qPCR. Bioinformatics analysis was performed to predict target miRNAs and mRNAs and construct a circRNA-miRNA-mRNA network. Pathway and process enrichment analyses were performed to explore the potential biological roles of DE circRNAs.

**Results:**

Berberine decreased GC cell viability, cell proliferation, and Δ*ψ*m and induced cell apoptosis. Thirty-one DE circRNAs were identified in the berberine-treated group compared to the control group, among which circRNA2499, hsa_circ_0003423, and hsa_circ_0006702 were validated using RT-qPCR. Enrichment analyses, based on the host genes of these 31 DE circRNAs and putative target mRNAs in the circRNA-miRNA-mRNA network of the validated circRNAs, indicated that berberine exerts anti-GC effects in multiple pathways including the Notch, MAPK, and NF-*κ*B signaling pathways via specific circRNAs.

**Conclusion:**

This study elucidated the expression profile of circRNAs in human GC cells after berberine treatment. Our results demonstrate that berberine has the potential to influence cancer-related pathways by regulating circRNA expression and their corresponding target genes in GC cells.

## 1. Introduction

Despite advancements in early diagnosis and therapeutics, such as surgery and chemoradiotherapy, the prognosis of gastric cancer (GC) remains relatively poor [1, 2]. Multiple factors including genetics and epigenetics are involved in the development of GC [3, 4]. Circular RNAs (circRNAs) are noncoding RNAs with a covalently closed continuous loop [5]. circRNAs can regulate gene expression by acting as nuclear transcriptional regulators, miRNA sponges, and RNA-binding protein sponges [6]. Many researchers have highlighted the important functions of circRNAs in the development of cancer, including hepatocellular carcinoma [7], GC [8], and colon cancer [9].

Natural products represent an important source for discovering anticancer agents. Several plant-derived agents have been successfully used in cancer treatment, such as vinca alkaloid, etoposide, and paclitaxel, and some others are currently under investigation [10, 11]. Some studies reported that natural products such as nitidine chloride [12] and quercetin [13] exert anticancer effects or improve the prognosis of patients by influencing the expression of circRNAs. Several studies have demonstrated the important anticancer roles of berberine against malignant tumors, including GC [14]. However, the mechanisms of berberine against GC via circRNAs remain unclear.

In this study, RNA-seq analysis was performed to identify and analyze changes in circRNAs in GC cells in response to berberine. These results improve the understanding of the circRNAs targeted by berberine, which may be useful in developing treatments for GC.

## 2. Materials and Methods

### 2.1. Cell and Drug Preparation

Human AGS and HGC27 GC cells were obtained from the Cell Bank of the Chinese Academy of Sciences (Shanghai, China) and maintained in F-12K (Gibco, Thermo Fisher Scientific; Waltham, USA) and RPMI 1640 (Gibco) media, respectively. Both media were supplemented with 10% fetal bovine serum (Gibco). The cells were maintained in a humidified atmosphere of 5% CO_2_ at 37°C. Berberine (MedChemExpress, Shanghai, China) was dissolved in dimethyl sulfoxide (Solarbio, Beijing, China) and diluted to the working concentration with culture media.

### 2.2. Cell Viability Assay

AGS and HGC27 cells were seeded into 96-well plates and incubated with berberine (0, 20, 50, and 80 *μ*M) for 24, 48, and 72 h. After incubation, cell viability was examined using a Cell Counting Kit-8 (CCK-8) kit (Dojindo, Kumamoto, Japan) by measuring the optical density values.

### 2.3. Cell Colony Formation Assay

AGS and HGC27 cells were seeded into 6-well plates and incubated with berberine (0 and 50 *μ*M). After 7 days in culture, cells were stained with crystal violet (Beyotime, Shanghai, China) and photographed with a digital camera. The colony formation rate was calculated, which can account for cell population dependence and proliferation ability.

### 2.4. Flow Cytometry Evaluation of Cell Apoptosis

AGS and HGC27 cells treated with berberine were harvested and cell apoptosis induced due to berberine was identified using the annexin V-FITC/PI (Beyotime, Shanghai, China) staining according to the instructions. The percentage of apoptotic cells was assessed using a FACSCalibur flow cytometer (BD Biosciences, San Jose, CA, USA).

### 2.5. Measurement of Mitochondrial Membrane Potential

AGS cells were cultured and treated with different concentrations of berberine in 24-well plates. Thereafter, the mitochondrial membrane potential (Δ*ψ*m) was determined using a JC-1 probe kit (MedChemExpress) according to the instructions. Red and green fluorescence in the cells was examined using a fluorescence microscope (Leica, Wetzlar, Germany). The ratio of red to green fluorescence intensity was calculated using ImageJ software (National Institutes of Health, Bethesda, MD, USA).

### 2.6. RNA Library Construction and circRNA Sequencing

Three random samples from each group were subjected to RNA-seq. Total RNA was isolated using TRIzol reagent (Invitrogen, Carlsbad, CA, USA) according to the instructions. RNA concentration and purity were quantified with a NanoDrop ND-1000 (Thermo Fisher Scientific). RNA integrity was assessed using an Agilent 2100 (Agilent Technologies, Santa Clara, CA, USA). Ribosomal RNA was depleted from total RNA according to the Ribo-Zero rRNA Removal Kit instructions (Illumina, San Diego, CA, USA). Preparation of RNA libraries and sequencing were conducted by LC Bio Co., Ltd. (Hangzhou, China). Sequencing was performed on a NovaSeq 6000 (Illumina) according to the instructions. Differentially expressed (DE) circRNAs were identified as those showing a |log2 (fold-change)| ≥ 1 and statistical significance (*P* value < 0.05) according to R package edgeR [15].

### 2.7. Pathway and Process Enrichment Analyses

The effects of circRNAs on their host genes were predicted using GO (http://www.geneontology.org) and KEGG (http://www.kegg.jp) enrichment analyses to explore meaningful gene annotations. The *P* values represent enrichment scores. GO analysis was performed to evaluate biological processes, cellular components, and molecular functions.

### 2.8. RT-qPCR Validation

Total RNA was isolated from the cell lines using TRIzol reagent (Invitrogen) and then reverse-transcribed into cDNA using the PrimerScript RT reagent kit with gDNA Eraser (Takara Bio, Shiga, Japan). RT-qPCR was performed using SYBR Premix Ex Taq (Takara), and *GAPDH* was used as an internal control. The expression of circRNAs was defined based on the threshold cycle (Ct), and relative expression levels were calculated using the 2^−ΔΔCt^ method. PCR amplification was performed as follows: 95°C for 30 s, 40 cycles of 95°C for 5 s, and 60°C for 34 s.

### 2.9. Construction of circRNA-miRNA-mRNA Network

Interactions of circRNA-miRNA and miRNA-mRNA, which were predicted using TargetScan (http://www.targetscan.org/) and miRanda (http://www.miranda.org/), were combined to construct a circRNA-miRNA-mRNA network. Cytoscape software v3.7.2 [16] was used to visualize the network. The Metascape database [17] provides reliable and productive bioinformatics analyses of gene or protein lists, which can help users to make better data-driven decisions. The predicted mRNAs were entered into the Metascape database for annotation, functional analysis, and MCODE algorithm [18] analysis.

### 2.10. Statistical Analyses

Experimental data are presented as the means ± SEM. Student's two-tailed unpaired *t*-test was used to evaluate the differences between the two groups. *P* values <0.05 represent statistical significance. GraphPad Prism 8.0 (http://www.graphpad.com) was used for statistical analyses.

## 3. Results

### 3.1. Berberine Decreased Cell Viability, Cell Proliferation, and Δ*ψ*m and Induced Cell Apoptosis

**Figure 1:**
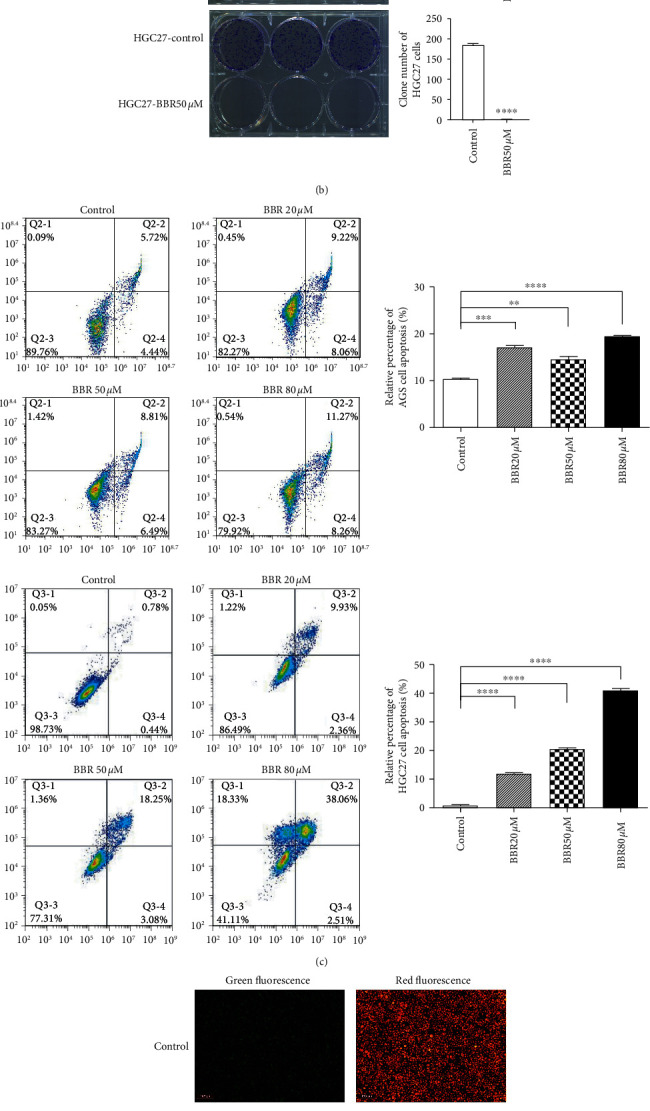
Anticancer effects of berberine (BBR) in gastric cancer cells. (a) AGS and HGC27 cell viability following berberine treatment for 24, 48, and 72 h determined using CCK-8 assay. (b) Colony formation assay showed the clone number in AGS and HGC27 cells treated with 50 *μ*M BBR or control. (c) AGS and HGC27 cell apoptosis after treatment with BBR for 48 h determined using flow cytometry. (d) Mitochondrial membrane potential of AGS cells following treatment with BBR for 48 h and staining with JC-1 probe. Data are presented as the means ± SEM; *P* value significance: 0 ≤ ^∗∗∗∗^ ＜ 0.0001 ≤ ^*∗∗∗*^ ＜ 0.001 ≤ ^*∗∗*^ ＜ 0.01 ≤ ^*∗*^ ＜ 0.05 ≤ ns; ns: not significant.

The CCK-8 analysis showed that AGS and HGC27 cell viability decreased in berberine concentration- and treatment time-dependent manners (0–80 *μ*M and 0–72 h, respectively) (Figure 1(a)). After 7 days of treatment, 50 *μ*M berberine significantly decreased AGS and HGC27 cell colony formation rate (Figure 1(b)). Treatment with different concentrations of berberine for 48 h resulted in an increased number of apoptotic AGS and HGC27 cells (Figure 1(c)), as well as a reduction in Δ*ψ*m in AGS cells (Figure 1(d)).

### 3.2. Overview of circRNA Profiles

**Figure 2:**
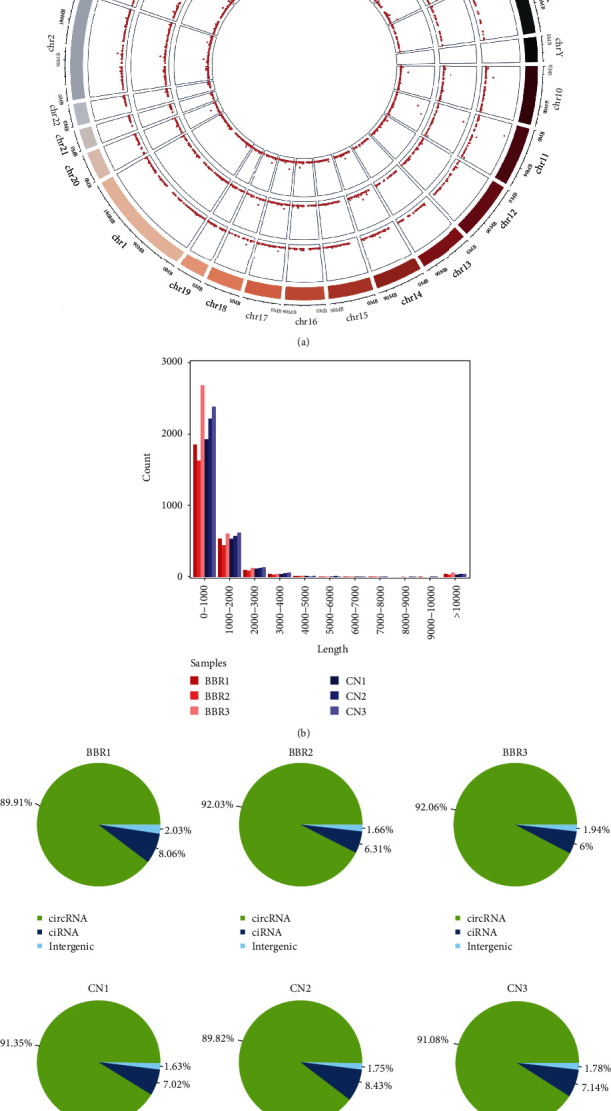
Overview of circular RNA (circRNA) profiles based on RNA-seq analysis. (a) Distribution of identified circRNAs on the human chromosome. (b) Length distribution of the circRNAs. (c) Genomic origin of the identified circRNAs (circRNA: exonic origin; ciRNA: intronic origin).

After 48 h treatment, 50 *μ*M berberine induced changes in the circRNA expression profiles. A total of 12,256 circRNAs were detected using RNA-seq analysis between berberine-treated and control AGS cells. Among these, 5561 potential novel circRNAs have not been identified previously.  Figure 2(a) represents the chromosome distribution of the identified circRNAs. No circRNAs were distributed in chromosome Y. The length distribution of all identified circRNAs is visualized in Figure 2(b). The lengths of the circRNAs ranged from <1000 nucleotides (nt) to >10,000 nt, with the majority having <1000 nt. The genomic origins of the identified circRNAs are presented in Figure 2(c). Based on their location in the genome, most candidate circRNAs' host genes were obviously derived from exonic regions, suggesting the importance of these circRNAs.

### 3.3. DE circRNAs Affected by Berberine

**Figure 3:**
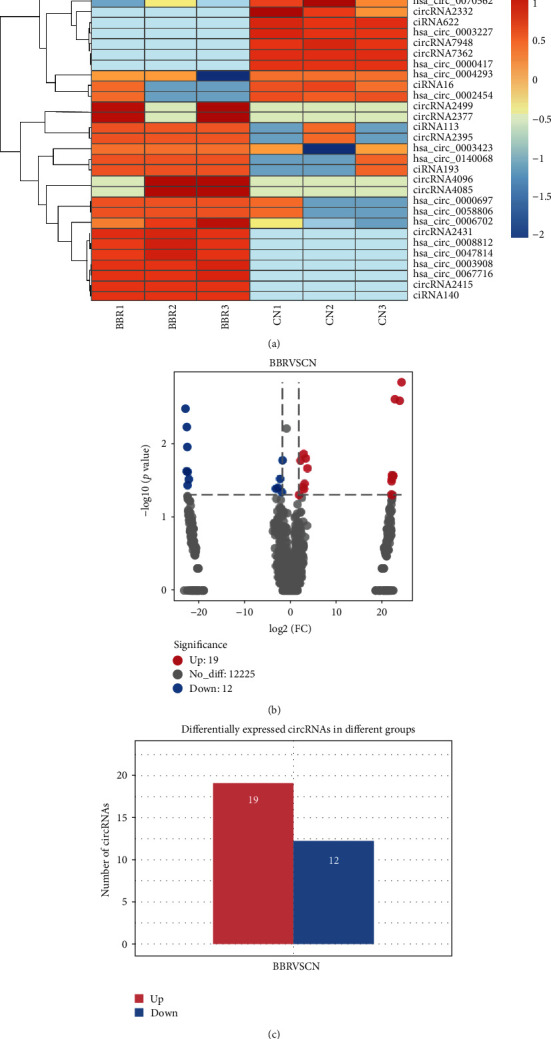
Differentially expressed (DE) circRNAs in AGS cells after treatment with berberine (BBR). (a) Clustered heatmap showing all DE circRNAs between the BBR-treated group and control group. (b) Volcano plot filtering identified DE circRNAs selected with |log2(fold change)| ≥ 1 and *P* value < 0.05. (c) Total number of up- and downregulated DE circRNAs.

Figure 3(a) shows a clustered heatmap of the DE circRNAs in the berberine-treated group and control group determined using the described cutoff. The volcano plot shows significant DE circRNAs (Figure 3(b)). We identified 31 DE circRNAs, among which 19 were upregulated and 12 were downregulated in the berberine-treated group (Figures 3(b) and 3(c)).

### 3.4. DE circRNA-Hosting Gene GO and KEGG Analyses

**Figure 4:**
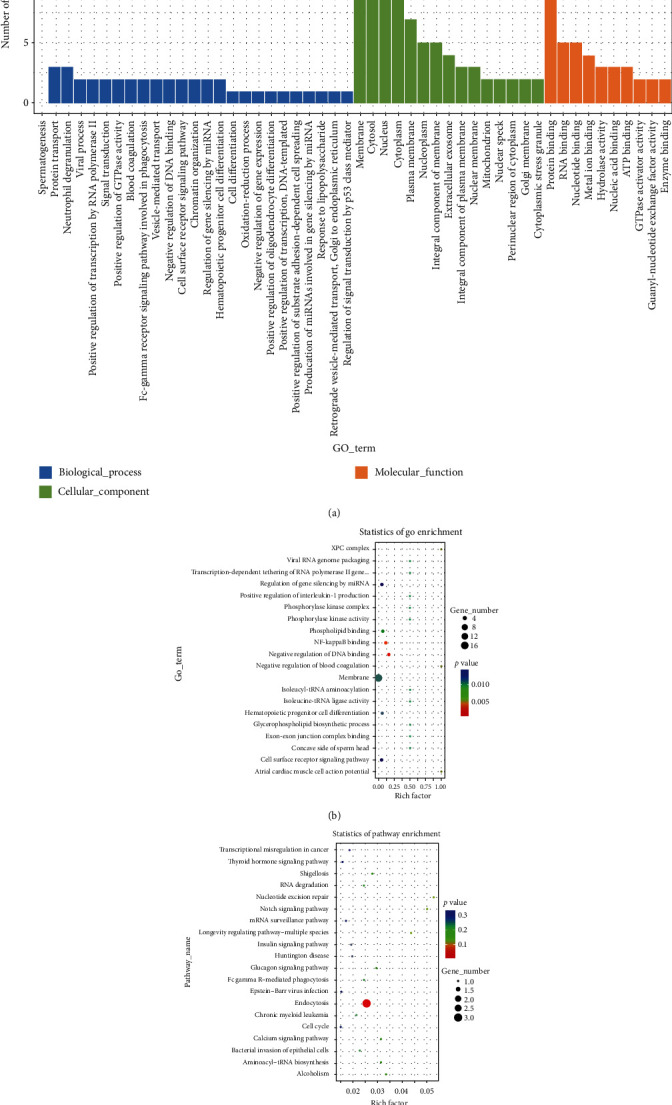
GO and KEGG pathway analyses of DE circRNA-hosting genes. (a) Representative GO terms of biological processes, cellular components, and molecular functions, (b) top 20 GO analyses, and (c) top 20 KEGG analyses.

To annotate the DE circRNAs, enrichment analysis was performed on their host genes. GO analysis (Figures 4(a) and 4(b)) revealed that the altered circRNAs were associated with terms such as negative regulation of DNA binding (GO:0043392), NF-*κ*B binding (GO:0051059), phospholipid binding (GO:0005543), regulation of interleukin-1 production (GO:0032732), and phosphorylase kinase activity (GO:0004689). KEGG analysis (Figure 4(c)) yielded enrichment in terms such as nucleotide excision repair (ko03420), Notch signaling pathway (ko04330), insulin signaling pathway (ko04910), and cell cycle (ko04110).

### 3.5. Validation of DE circRNAs

**Figure 5:**
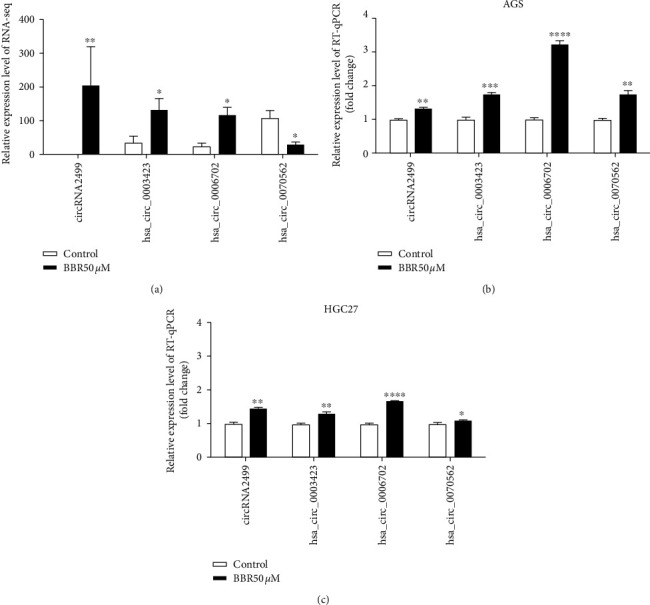
Validation of DE circRNAs. (a) Four circRNAs with significantly different expression levels between the berberine-treated group and the control group in AGS cells, which were assessed via the RNA-seq approach. (b, c) RT-qPCR validation of DE circRNAs in AGS and HGC27 cells. Data are presented as means ± SEM. *P* value significance: 0 ≤ ^*∗∗∗∗*^ ＜ 0.0001 ≤ ^*∗∗∗*^ ＜ 0.001 ≤ ^*∗∗*^ ＜ 0.01 ≤ ^*∗*^ ＜ 0.05.

To verify the RNA-seq results, four DE circRNAs were manually selected at random for further validation by RT-qPCR in AGS and HGC27 cells. The primer sequences for the candidate circRNAs are shown in Table S1. The expression levels of circRNA2499 (1.33-fold; *P*<0.01), hsa_circ_0003423 (1.76-fold; *P*<0.001), hsa_circ_0006702 (3.25-fold; *P*<0.0001), and hsa_circ_0070562 (1.76-fold; *P*<0.01) were significantly upregulated in the berberine-treated group of AGS cells. The expression levels of circRNA2499 (1.47-fold; *P* < 0.01), hsa_circ_0003423 (1.31-fold; *P* < 0.01), hsa_circ_0006702 (1.69-fold; *P* < 0.0001), and hsa_circ_0070562 (1.09-fold; *P* < 0.05) were also significantly upregulated in the berberine-treated group of HGC27 cells. The RT-qPCR results of circRNA2499, hsa_circ_0003423, and hsa_circ_0006702 in AGS and HGC27 cells agreed with those of RNA-seq (Figure 5).

### 3.6. circRNA-miRNA-mRNA Network and Biological Function Prediction for Validated circRNAs

**Figure 6:**
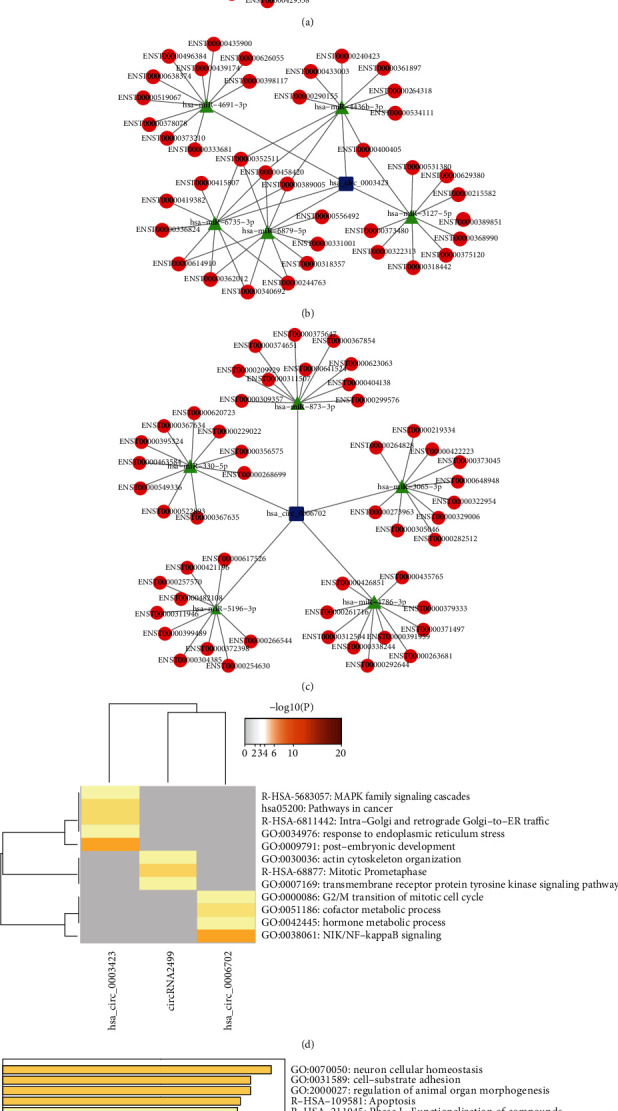
circRNA-miRNA-mRNA network and biological function prediction of validated circRNAs. circRNA-miRNA-mRNA regulation networks of (a) circRNA2499, (b) hsa_circ_0003423, and (c) hsa_circ_0006702 visualized using Cytoscape v3.7.2. Enrichment analysis of (d) target genes of each circRNA and (e) merged target genes of the three circRNAs using Metascape database.

To estimate the functions of circRNA2499, hsa_circ_0003423, and hsa_circ_0006702, the circRNAs were assumed to act as miRNA sponges and take part in a circRNA-miRNA-mRNA network. The top five miRNAs predicted to bind to the circRNAs as well as each miRNA's top ten target mRNAs are shown in Figures 6(a)–6(c). The target mRNAs represented by their transcript IDs in Figures 6(a)–6(c) are listed in Supplementary Table S2.

To further evaluate the circRNAs, enrichment analysis based on putative target mRNAs was conducted and the results were visualized using the Metascape database. Analysis of each circRNA (Figure 6(d) and Table S3) indicated that the target genes of circRNA2499 were associated with terms such as mitotic prometaphase (R-HSA-68877); target genes of hsa_circ_0003423 were associated with terms such as MAPK family signaling cascades (R-HSA-5683057) and response to endoplasmic reticulum stress (GO:0034976); and target genes of hsa_circ_0006702 were associated with terms such as NIK/NF-*κ*B signaling (GO:0038061), hormone metabolic process (GO:0042445), and G2/M transition of the mitotic cell cycle (GO:0000086). In addition, the merged target genes of these three circRNAs (Figure 6(e) and Table S4) were associated with terms such as cell-substrate adhesion (GO:0031589), apoptosis (R-HSA-109581), and PTEN regulation (R-HSA-6807070). MCODE module analysis using the Metascape database showed that the target mRNAs were mainly enriched in the biological process of cell cycle (Table 1).

## 4. Discussion

The use of berberine, a compound isolated from medicinal plants such as *Coptis chinensis*, has been reported for preventing and treating GC via targeting the AMPK/HNF4*α*/WNT5A pathway [19], Akt/mTOR/p70S6/S6 pathway [20], interleukin-8 [21], and survivin and STAT3 [22]. Our study focused on the effects of berberine on circRNA expression profiles in human GC cells.

After observing berberine-induced changes in GC cell viability, cell colony formation rate, cell apoptosis, and Δ*ψ*m, the circRNA expression alterations in AGS cells treated with berberine were compared with those in the control group. The length of most circRNAs was 0–1000 nt, which is consistent with the findings of a previous study [23]. CircRNAs are mainly derived from exons or introns of their host linear transcripts and are involved in regulating their host gene expression [24, 25]. Accordingly, after screening 31 DE circRNAs induced by berberine, the annotations of their host genes were estimated by GO and KEGG analyses. Numerous pathways have been demonstrated to exert important effects in the mechanisms of GC onset and development, including interleukin-1 [26], NF-*κ*B [27], and the Notch signaling pathway [28].

Analyses of host genes cannot completely reveal the roles of circRNAs. circRNAs can also function as miRNA sponges [29]. In this study, such circRNAs (circRNA2499, hsa_circ_0003423, and hsa_circ_0006702) were identified and further analyzed. circRNA2499 is a newly predicted circRNA in our current RNA-seq analysis. circRNA-miRNA-mRNA networks with these validated circRNAs were constructed to predict their biological functions. Several functions were reported to be closely associated with GC, including cell cycle, cell-substrate adhesion, apoptosis, and the NF-*κ*B and MAPK signaling pathways.

In enrichment analyses, cell apoptosis and mitochondrion alterations induced by berberine were validated in our study. Several crucial regulators of apoptosis maintain or destroy the integrity of the mitochondrial membrane [30]. Our results indicate that berberine can reduce Δ*ψ*m in AGS cells. NF-*κ*B is one of the most important transcription factors linking chronic inflammation and cancer and is activated in cancer cells and the tumor microenvironments of most cancers [31]. Previous reports demonstrated that berberine can suppress NF-*κ*B expression in gastric [32], colon [33], lung [34], breast [35], and prostate [36] cancer cells to exert anticancer effects. The MAPK family signaling cascade, which includes JNK, p38 MAPK, and ERK, is one of the main intracellular pathways for apoptosis [37]. It has been reported that berberine can induce colon cancer cell apoptosis through continuous phosphorylation of JNK and p38 MAPK [38], whereas another study reported that berberine inhibits GC cell growth by inactivating the p38/JNK pathway [32]. Our findings indicate that circRNA-miRNA-mRNA regulatory interactions play important roles in the treatment of GC with berberine.

Our study provides novel insight into the treatment of GC using berberine. Nevertheless, the animal and clinical assays are needed to investigate the regulatory relationships between DE circRNAs and their target miRNAs and mRNAs.

## 5. Conclusions

To summarize, circRNA sequencing analysis was conducted on berberine-treated and untreated AGS cells after investigating the appropriate concentrations and treatment durations for berberine to exert its anti-GC effects. The results, for the first time, demonstrated that berberine may influence cancer-related pathways by regulating circRNA expression and provided novel understandings of the mechanisms of berberine treatment for GC. The identified circRNAs, such as circRNA2499, hsa_circ_0003423, and hsa_circ_0006702, could be potential targets for GC treatment. Further studies are needed to evaluate the precise functions of these DE circRNAs in GC.

## Figures and Tables

**Table 1:** MCODE module enrichment analysis of merged target genes in three validated circRNAs in the circRNA-miRNA-mRNA network based on the Metascape database.

Term	Category	Description	Log10 (*P*)
R-HSA-69278	Reactome gene sets	Cell cycle, mitotic	−6.1
R-HSA-68877	Reactome gene sets	Mitotic prometaphase	−5.8
R-HSA-1640170	Reactome gene sets	Cell cycle	−5.6

## Data Availability

The datasets used and analyzed during the current study are available from the corresponding author on reasonable request.

## Supplementary Materials

Supplementary Materials Table S1: primers used for RT-qPCR assay. Table S2: mRNA names corresponding to transcript IDs. Table S3: enrichment analysis of target genes of each validated circRNA in the circRNA-miRNA-mRNA network based on the Metascape database. Table S4: enrichment analysis of the merged target genes of three validated circRNAs in the circRNA-miRNA-mRNA network based on the Metascape database
