# Supplementary material for: Effects of Berberine on Circular RNA Expression Profiles in Human Gastric Cancer Cells
Source: Evid Based Complement Alternat Med. 2021 May 4;2021:6688629. doi: 10.1155/2021/6688629 (PMC8112944; doi:10.1155/2021/6688629)
Supplement: Supplementary Materials — Table S1: primers used for RT-qPCR assay. Table S2: mRNA names corresponding to transcript IDs. Table S3: enrichment analysis of target genes of each validated circRNA in the circRNA-miRNA-mRNA network based on the Metascape database. Table S4: enrichment analysis of the merged target genes of three validated circRNAs in the circRNA-miRNA-mRNA network based on the Metascape database [file 6688629.f1.doc]

Table S1. Primers used for RT-qPCR assay

| **ID** | **Forward and reverse primers** | **Product length (base pairs, bp)** |
| --- | --- | --- |
| circRNA2499 | F: 5’-TGTGACCAACTCCACCTACG-3’ | 374 bp |
|  | R: 5’-CATGGACTACGTGCTTGTGG-3’ |  |
| hsa_circ_0003423 | F: 5’-CCTTGCTGTATCGCCACTT-3’ | 575 bp |
|  | R: 5’-GTGATGTGCTCCTCTCAACCA-3’ |  |
| hsa_circ_0006702 | F: 5’-TTGTCCTGGCAAGTGATGG-3’ | 463 bp |
|  | R: 5’-TTGTTACTGGACACGATGACC-3’ |  |
| hsa_circ_0070562 | F: 5’-GACCATAAGGCTCTTACTCTCA-3’ | 397 bp |
|  | R: 5’-CAGGTAAGGACTATGGTAGTGG-3’ |  |

Table S2. mRNA names corresponding to transcript IDs

| **Transcript ID** | **Gene name** |
| --- | --- |
| ENST00000209929 | FMO2 |
| ENST00000215582 | MISP |
| ENST00000216124 | ARSA |
| ENST00000217244 | CSNK2A1 |
| ENST00000218147 | BCORL1 |
| ENST00000219334 | SMPD3 |
| ENST00000229022 | VDR |
| ENST00000240423 | NCAPH |
| ENST00000244763 | SSR1 |
| ENST00000251547 | FBXO44 |
| ENST00000254630 | PTCD3 |
| ENST00000257570 | OASL |
| ENST00000260364 | NOX5 |
| ENST00000261716 | TAOK1 |
| ENST00000262269 | MYH14 |
| ENST00000263334 | PAX8 |
| ENST00000263335 | PAX8 |
| ENST00000263681 | POLD3 |
| ENST00000263798 | TYRO3 |
| ENST00000264039 | GPC1 |
| ENST00000264318 | GABRA4 |
| ENST00000264828 | COL5A3 |
| ENST00000266544 | NDUFA9 |
| ENST00000268699 | GAS8 |
| ENST00000273963 | KLHL8 |
| ENST00000282512 | NADK2 |
| ENST00000290155 | CFAP298 |
| ENST00000292644 | PSMC2 |
| ENST00000298351 | TMEM63C |
| ENST00000299576 | AKIP1 |
| ENST00000304385 | TMEM154 |
| ENST00000305046 | ADH1B |
| ENST00000308527 | SNX33 |
| ENST00000309357 | AKIP1 |
| ENST00000311507 | PLAC8 |
| ENST00000311623 | ODAPH |
| ENST00000311946 | NIPAL4 |
| ENST00000312504 | VWC2L |
| ENST00000317233 | AFF3 |
| ENST00000318357 | ANKRD23 |
| ENST00000318442 | ZNF366 |
| ENST00000322313 | WDR33 |
| ENST00000322954 | UACA |
| ENST00000329006 | MANEAL |
| ENST00000331001 | ANKRD23 |
| ENST00000333681 | BCL2 |
| ENST00000336824 | FNDC3B |
| ENST00000338244 | SLC23A2 |
| ENST00000339266 | DLGAP4 |
| ENST00000340692 | MOCS1 |
| ENST00000342301 | POU2F2 |
| ENST00000348715 | PAX8 |
| ENST00000352511 | ACVR2B |
| ENST00000356575 | MEGF6 |
| ENST00000359209 | KIRREL1 |
| ENST00000360032 | DMBX1 |
| ENST00000361897 | NOS1AP |
| ENST00000362012 | PTGS1 |
| ENST00000366932 | RRP15 |
| ENST00000366977 | NSL1 |
| ENST00000367634 | RALGPS2 |
| ENST00000367635 | RALGPS2 |
| ENST00000367854 | RCSD1 |
| ENST00000368172 | KIRREL1 |
| ENST00000368173 | KIRREL1 |
| ENST00000368990 | PLEKHA1 |
| ENST00000371497 | TSHZ2 |
| ENST00000372398 | NCS1 |
| ENST00000373045 | MANEAL |
| ENST00000373210 | AGO4 |
| ENST00000373218 | EIF4EBP2 |
| ENST00000373480 | KIAA1522 |
| ENST00000373913 | DLGAP4 |
| ENST00000374651 | ZBTB40 |
| ENST00000375120 | OTUD3 |
| ENST00000375647 | ZBTB40 |
| ENST00000376970 | MYH14 |
| ENST00000378078 | RGP1 |
| ENST00000379333 | SLC23A2 |
| ENST00000389005 | NCBP3 |
| ENST00000389851 | UVSSA |
| ENST00000391959 | PPP1R12B |
| ENST00000395324 | VDR |
| ENST00000396251 | XIRP1 |
| ENST00000397647 | PAX8 |
| ENST00000398117 | BCL2 |
| ENST00000399489 | HMGB1 |
| ENST00000400405 | SIAH3 |
| ENST00000401952 | DLGAP4 |
| ENST00000404138 | ZBTB40 |
| ENST00000409236 | AFF3 |
| ENST00000415807 | FNDC3B |
| ENST00000419482 | SMYD1 |
| ENST00000421196 | AKR1C2 |
| ENST00000422223 | FREM1 |
| ENST00000425460 | MYH14 |
| ENST00000426851 | ZNF398 |
| ENST00000429538 | PAX8 |
| ENST00000433003 | MED14 |
| ENST00000435765 | PSMC2 |
| ENST00000435900 | PISD |
| ENST00000439174 | GNA13 |
| ENST00000456822 | BCORL1 |
| ENST00000458420 | CLSTN2 |
| ENST00000463584 | PFDN6 |
| ENST00000482108 | PEG10 |
| ENST00000496384 | BRAF |
| ENST00000519067 | SLC45A4 |
| ENST00000522893 | ERICH1 |
| ENST00000531380 | FZD4 |
| ENST00000534111 | TMPRSS4 |
| ENST00000549336 | VDR |
| ENST00000556492 | NAA30 |
| ENST00000560215 | GLDN |
| ENST00000579991 | DYNLL2 |
| ENST00000601313 | MYH14 |
| ENST00000609713 | KCNJ6 |
| ENST00000610020 | RPAP2 |
| ENST00000612912 | TJAP1 |
| ENST00000614910 | PTGS1 |
| ENST00000615002 | TEK |
| ENST00000616557 | ODAPH |
| ENST00000617335 | MTX3 |
| ENST00000617526 | PEG10 |
| ENST00000620723 | GAS8 |
| ENST00000623063 | ADSL |
| ENST00000626055 | ADH5 |
| ENST00000629380 | SYNGAP1 |
| ENST00000638374 | GOSR2 |
| ENST00000641524 | OR4E2 |
| ENST00000642316 | MYH14 |
| ENST00000646561 | CSNK2A1 |
| ENST00000648948 | TRAPPC9 |

Table S3. Enrichment analysis of target genes of each validated circRNA in the circRNA-miRNA-mRNA network based on the Metascape database

| **ID** | **Term** | **Category** | **Description** | **Log10(P)** |
| --- | --- | --- | --- | --- |
| hsa_circ_0006702 | GO:0038061 | GO Biological Processes | NIK/NF-kappa B signaling | –3.65 |
| hsa_circ_0003423 | GO:0009791 | GO Biological Processes | Post-embryonic development | –3.61 |
| circRNA2499 | R-HSA-68877 | Reactome Gene Sets | Mitotic prometaphase | –2.79 |
| hsa_circ_0003423 | hsa05200 | KEGG Pathway | Pathways in cancer | –2.62 |
| hsa_circ_0006702 | GO:0051186 | GO Biological Processes | Cofactor metabolic process | –2.57 |
| hsa_circ_0003423 | R-HSA-6811442 | Reactome Gene Sets | Intra-Golgi and retrograde Golgi-to-ER traffic | –2.52 |
| hsa_circ_0006702 | GO:0042445 | GO Biological Processes | Hormone metabolic process | –2.46 |
| hsa_circ_0006702 | GO:0000086 | GO Biological Processes | G2/M transition of mitotic cell cycle | –2.12 |
| circRNA2499 | GO:0030036 | GO Biological Processes | Actin cytoskeleton organization | –2.12 |
| hsa_circ_0003423 | R-HSA-5683057 | Reactome Gene Sets | MAPK family signaling cascades | –2.11 |
| hsa_circ_0003423 | GO:0034976 | GO Biological Processes | Response to endoplasmic reticulum stress | –2.07 |
| circRNA2499 | GO:0007169 | GO Biological Processes | Transmembrane receptor protein tyrosine kinase signaling pathway | –2.02 |

Table S4. Enrichment analysis of the merged target genes of three validated circRNAs in the circRNA-miRNA-mRNA network based on the Metascape database

| **Term** | **Category** | **Description** | **Log10(P)** |
| --- | --- | --- | --- |
| GO:0070050 | GO Biological Processes | Neuron cellular homeostasis | –3.41 |
| GO:0031589 | GO Biological Processes | Cell-substrate adhesion | –3.14 |
| GO:2000027 | GO Biological Processes | Regulation of animal organ morphogenesis | –3.14 |
| R-HSA-109581 | Reactome Gene Sets | Apoptosis | –3.01 |
| R-HSA-211945 | Reactome Gene Sets | Phase I - Functionalization of compounds | –2.98 |
| GO:0046660 | GO Biological Processes | Female sex differentiation | –2.83 |
| R-HSA-68877 | Reactome Gene Sets | Mitotic prometaphase | –2.77 |
| GO:0010817 | GO Biological Processes | Regulation of hormone levels | –2.72 |
| R-HSA-6807070 | Reactome Gene Sets | PTEN Regulation | –2.54 |
| GO:0019748 | GO Biological Processes | Secondary metabolic process | –2.52 |
| GO:0048705 | GO Biological Processes | Skeletal system morphogenesis | –2.47 |
| GO:0008643 | GO Biological Processes | Carbohydrate transport | –2.42 |
| GO:0007519 | GO Biological Processes | Skeletal muscle tissue development | –2.35 |
| R-HSA-5683057 | Reactome Gene Sets | MAPK family signaling cascades | –2.11 |
| R-HSA-196854 | Reactome Gene Sets | Metabolism of vitamins and cofactors | –2.08 |
| GO:0006367 | GO Biological Processes | Transcription initiation from RNA polymerase II promoter | –2.07 |
